# First report of Y-linked genes in the kissing bug *Rhodnius prolixus*

**DOI:** 10.1186/s12864-016-2425-8

**Published:** 2016-02-09

**Authors:** Leonardo B Koerich, Eduardo G Dupim, Leonardo L Faria, Felipe A Dias, Ana F Dias, Gabriela S Trindade, Rafael D Mesquita, Antonio B Carvalho

**Affiliations:** Departamento de Genética, Instituto de Biologia, Universidade Federal do Rio de Janeiro, Rio de Janeiro, Rio de Janeiro Brazil; Departamento de Parasitologia, Instituto de Ciencias Biologicas, Universidade Federal de Minas Gerais, Belo Horizonte, Minas Gerais Brazil; Instituto de Bioquímica Médica, Universidade Federal do Rio de Janeiro, Rio de Janeiro, Rio de Janeiro Brazil; Departamento de Bioquímica, Instituto de Química, Universidade Federal do Rio de Janeiro, Rio de Janeiro, Rio de Janeiro Brazil

**Keywords:** *Rhodnius prolixus*, Y chromosome, genomics, metalloproteinase, iron biding regulatory protein, zinc finger protein, testis, Y-linkage

## Abstract

**Background:**

Due to an abundance of repetitive DNA, the annotation of heterochromatic regions of the genome such as the Y chromosome is problematic. The Y chromosome is involved in key biological functions such as male-fertility and sex-determination and hence, accurate identification of its sequences is vital. The hemipteran insect *Rhodnius prolixus* is an important vector of Chagas disease, a trypanosomiasis affecting 6–7 million people worldwide. Here we report the identification of the first Y-linked genes of this species.

**Results:**

The *R. prolixus* genome was recently sequenced using separate libraries for each sex and the sequences assembled only with male reads are candidates for Y linkage. We found 766 such candidates and PCR tests with the ten largest ones, confirmed Y-linkage for all of them, suggesting that "separate libraries" is a reliable method for the identification of Y-linked sequences. BLAST analyses of the 766 candidate scaffolds revealed that 568 scaffolds contained genes or part of putative genes. We tested Y-linkage for 36 candidates and found that nine of them are Y-linked (the PCR results for the other 25 genes were inconclusive or revealed autosomal/X-linkage). Hence, we describe in this study, for the first time, Y-linked genes in the R. prolixus genome: two zinc finger proteins (*Znf-Y1* and *Znf-Y2*), one metalloproteinase (*Met-Y*), one aconitase/iron regulatory protein (*Aco-Y*) and five genes devoid of matches in any database (*Rpr-Y1* to *Rpr-Y5*). Expression profile studies revealed that eight genes are expressed mainly in adult testis (some of which presented a weak expression in the initial developmental stages), while *Aco-Y* has a gut-restricted expression.

**Conclusions:**

In this study we showed that the approach used for the *R. prolixus* genome project (separate sequencing of male and female DNA) is key to easy and fast identification of sex-specific (e.g. Y chromosome sequences). The nine new *R. prolixus* Y-linked genes reported here provide unique markers for population and phylogenetic analysis and further functional studies of these genes may answer some questions about sex determination, male fertility and Y chromosome evolution in this important species.

**Electronic supplementary material:**

The online version of this article (doi:10.1186/s12864-016-2425-8) contains supplementary material, which is available to authorized users.

## Background

Y chromosomes are widespread in plants and animals and are directly involved with important biological phenomena, such as sex-determination and male fertility [1]. The Y chromosome is heterochromatic in most species, hindering the identification of Y-linked sequences in many genome projects [2–5]. The best known Y chromosomes are the mammalian (human, chimp and macaque) and the fruit fly (Drosophila) chromosomes, in which a vast investment of time and resources resulted in a fairly accurate knowledge of their gene content [6–11]. Most genome projects rely on the Whole Genome Shotgun approach (WGS) in which euchromatic portions of the genome are assembled in large (and usually mapped) scaffolds, while heterochromatic regions are scattered in small unmapped scaffolds [5]. Heterochromatic genes suffer with this assembly problem and in most cases their exons are assembled in separate scaffolds [2, 3, 11].

A successful approach to find Y-linked genes using a combination of computational and experimental methods was demonstrated by Carvalho and coworkers [10, 11]. However, the process was labor intensive and new approaches have been proposed to improve the identification of Y-linked sequences. Recently, two independent studies showed that shallow sequencing of males and female can be used to identify Y-linked sequences in previously assembled genomes. In the first study, Hall and co-workers did Illumina sequencing of male and female DNA and were able to identify six novel Y-linked genes in *Anopheles* mosquitoes [12] (this method was also used to describe the male determining gene in *Aedes aegypti* [13]). In the second study, Carvalho and Clark [14] sequenced female DNA and used it to find male specific sequences in the assembled genomes of *Drosophila virilis* and human. They were able to identify four new Y-linked genes in the *Drosophila virilis* genome and 300 kb of previously unidentified sequences in the human Y chromosome. Arguably the most direct and simplest method would be to sequence male and female DNA libraries separately; sequences assembled only with male reads are likely to be part of the Y chromosome. This method was proposed by Krzywinski and coworkers [15]; its main limitation being that its use must be decided prior to the start of the sequencing which may be problematic since Y chromosome and other hetrochromatic regions are not the main target of most genome sequencing projects. One of the goals of the *Rhodnius* Genome Project was to identify Y-linked genes, so from the begining it employed separate male and female libraries.

The triatomine *Rhodnius prolixus* (Hemiptera, Heteroptera) is a major vector of Chagas’ disease, a serious human tropical neglected disease present in Latin America [16, 17], caused by the unicellular parasite *Trypanosoma cruzi*. These insects have holocentric chromosomes characterized by the presence of a diffuse or non-localized centromere [18]. In spite of its biological importance, triatomine genetic studies are mostly limited to karyotyping. The number of sex-chromosomes and autosomes varies among triatomine species; the number autosomes (A) varies from 18 to 22 (being 20A the most common), and the number of X chromosomes ranging from XY to XXXY [19]. *R. prolixus* has 20 autosomes, a XY chromosome system and very little else is known about its chromosomes and sex-determination [19–21]. It was found that *Rhodnius* C-bands are minute, suggesting that heterochromatin is not a major component of this insect genome [20]. However, the *R. prolixus* Y chromosome is entirely heterochromatic [20]. The genome of *R. prolixus* was assembled from reads produced from separate male and female DNA libraries [22] as initially proposed by Krzywinski and coworkers [15]. Using the assembly information, we identified scaffolds assembled exclusively with male reads as Y candidates. Therefore, in this study we report the identification of the first nine new Y-linked genes in the *R. prolixus* genome. We also identified other 21 Y-linked sequences which may now be used as Y chromosome specific PCR markers. Further studies of the function of *R. prolixus* Y-linked genes may help in the understanding of the Y-chromosome evolution, its role in sex determination, reproductive behavior and male fertility.

## Results

### The *Rhodnius prolixus* genome data and identification of Y-linked candidates

For the *R. prolixus* genome project, male and female DNA were sequenced separately, by Sanger and 454 platforms, and assembled together [22]. The assembly used for this study (RproC1) is composed of 27,872 scaffolds, totalizing approximately 700 Mb [22] with N50 length of 848 kb (accession ACPB00000000.3). For the reason that sequences were produced from adult insects, we filtered the scaffolds for bacterial DNA. A total of 155 scaffolds (totalizing 310 Mb) have strong blast hits to bacterial DNA and were excluded.

**Fig. 1:**
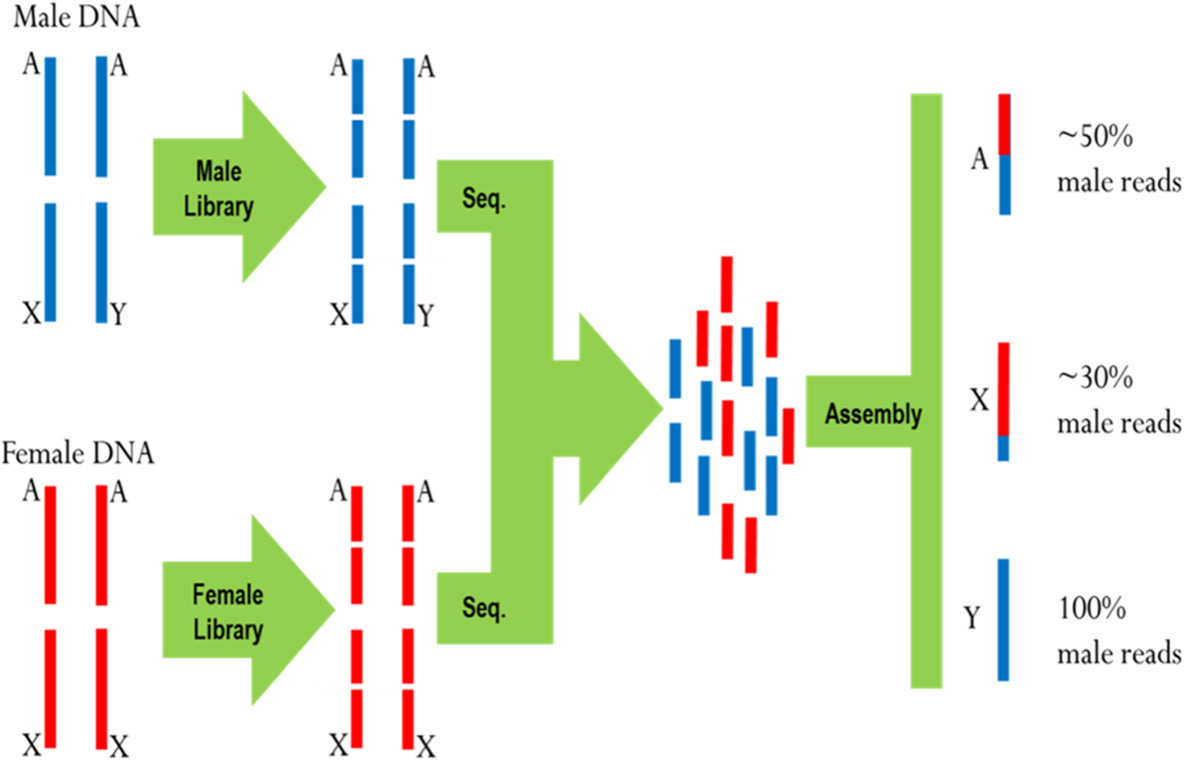
*R. prolixus* genome sequencing and assembly. Male and female DNAs were sequenced separately. This approach allowed us to count the number of reads from male libraries, which were then used in the assembly of each scaffold. Autosomal chromosomes (**a**) are equally represented in males and females (1:1); X-chromosomes are found in a ratio of 1 male for 2 female chromosomes (1:2); while Y chromosomes are found only in males (1:0)

Since genome libraries were built separately for each sex, we were able to identify the origin (male vs. female) of each read used to assemble each scaffold. Autosomal scaffolds are expected to be composed from equal numbers of male and female reads; whereas Y-linked candidates are composed only of male reads (Fig. 1). It is important to note that even X-linked scaffolds could be predicted using this approach, since we expect that such sequences are composed 2/3 of female reads (Fig. 1). However, we also expect a significant number of autosomes to be assembled with 2/3 of female reads. Such overlap (autosomes and X-linked sequences with equal number of female reads) should generate a substantial number of X-linked false positive candidates, making the identification of X-linked sequences more challenging.

To improve the reliability of our approach, we removed from our analysis all scaffolds that were assembled with less than 10 reads; since there is a higher chance of finding false positives in scaffolds assembled with a small number of reads (see Additional file 1: Text and Additional file 2: Table S1). After the removal of scaffolds built with 10 reads or less, a total of 10,512 scaffolds remained for further analysis. These scaffolds encompass 679.7 Mb and have an N50 length of 911 kb.

**Fig. 2:**
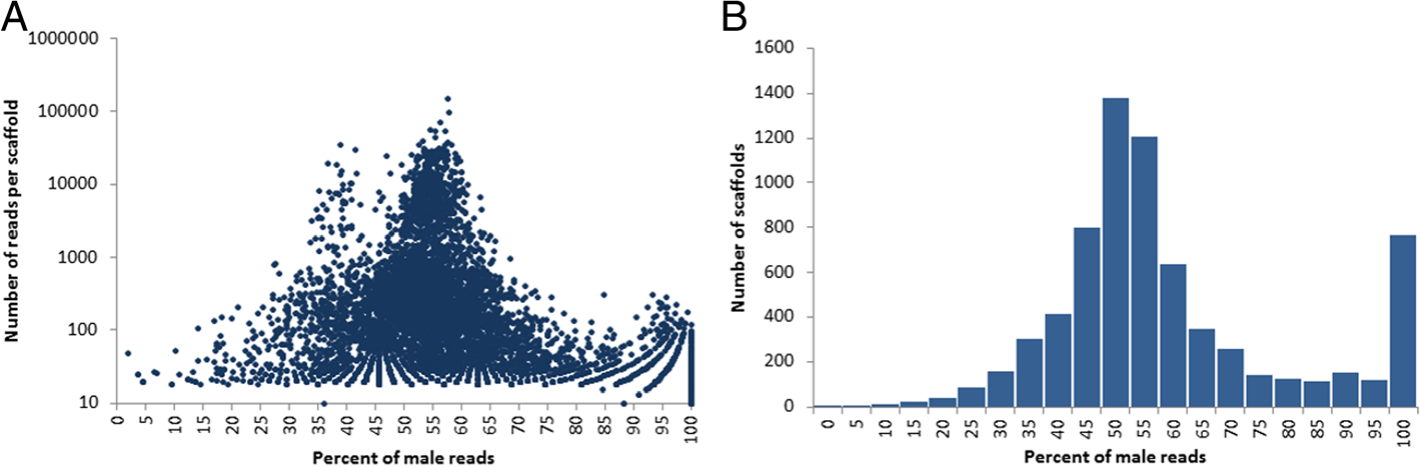
Proportion of male reads for each scaffold. A computer program was developed in order to count the number of reads from male libraries in each scaffold. The information produced was plotted according to: (**a**) the percentage of male reads and scaffold size or; (**b**) the sum of scaffolds for each percentage of male reads. Scaffolds composed of male reads only (100 %) were considered Y-linked candidates

**Fig. 3:**
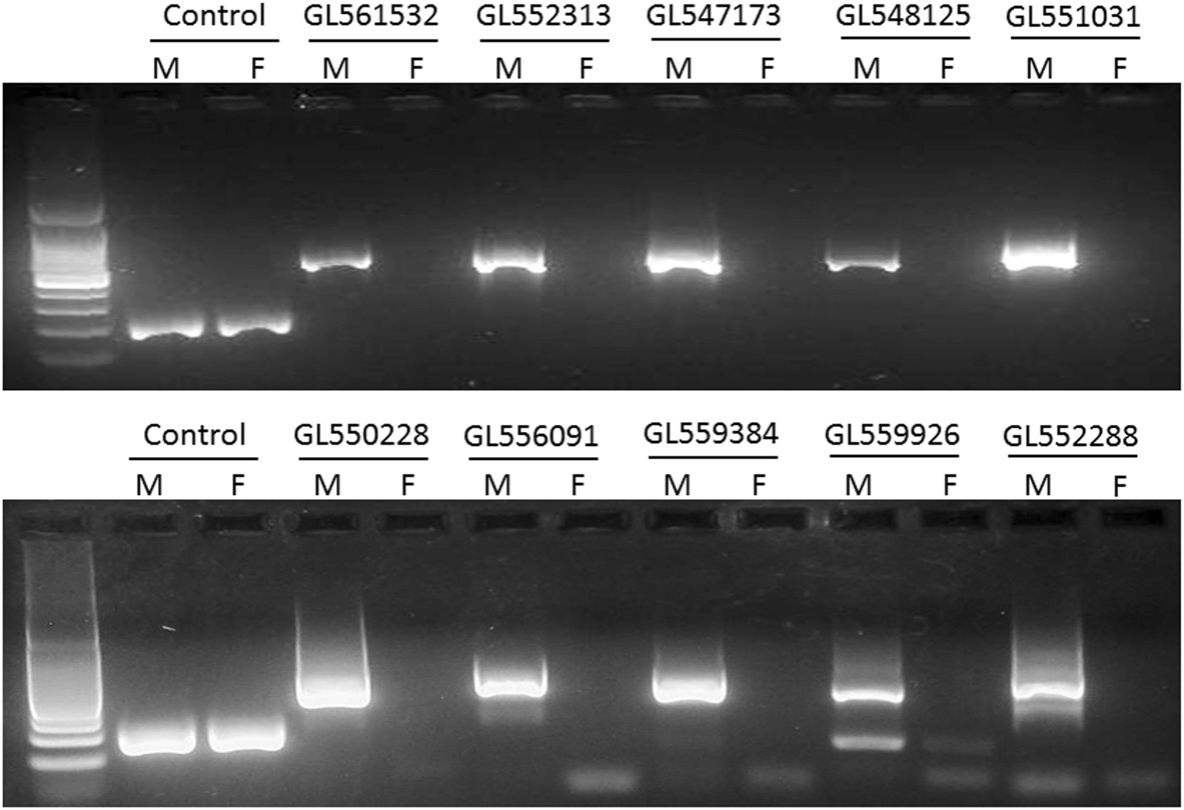
Scaffold linkage tests for the *Rhodinius prolixus* Y-chromosome. Y-linkage was confirmed by PCR. A male specific band implies Y-chromosome linkage. We tested the 10 largest Y-candidates and confirmed Y-linkage for all of them. Primers targeting scaffold GL563091 (61 % male reads) were used as control

We found 766 candidate scaffolds that were assembled only with male reads and these were considered as candidates for linkage to the *Rhodnius* Y chromosome (Fig. 2). We proceeded to select the ten largest scaffolds for Y-linkage tests as a preliminary test of accuracy for our approach. PCR results showed that all ten scaffolds are indeed Y-linked (Fig. 3), showing that the method is accurate for large scaffolds. Subsequent Y-linkage tests in randomly selected scaffolds revealed similar accuracy of Y-linkage prediction (11 out of 13, Additional file 3: Figure S1). As a result, we concluded with 30 experimentally confirmed Y-linked scaffolds (21 scaffolds from our initial tests plus the nine scaffolds containing Y-linked genes) in the *R. prolixus* genome (Additional file 4: Table S2).

### Identification of Y-linked genes

All 766 Y-candidate scaffolds were first softmasked with RepeatMasker to avoid repetitive sequences (i.e. transposable elements, virus retrogenes and simple repeats). Softmasked sequences were then blasted against different databases (NCBI Reference Sequences - RefSeq; NCBI Non-Redundant Proteins - nr; *R. prolixus* ESTs; *R. prolixus* annotated genes – GeneID and a set of *R. prolixus* predicted incomplete genes – GeneID-Trash). A total of 568 scaffolds presented hits in one or more databases. From these, only 47 have similarity with RefSeq proteins and a total of 106 scaffolds seemed to contain at least a part of putative protein from the NCBI nr database. A total of 458 scaffolds have blast hits only against *R. prolixus* transcripts and/or annotated genes (complete or partially annotated) and some of these could be novel proteins. Based on different blast evidences (see Materials and Methods section), we selected 36 scaffolds (each containing one gene or a piece of a gene) to test for Y-linkage. We found that nine genes in nine different scaffolds were Y-linked. Among the remaining 27 candidates, nine produced inconclusive results (PCR failed or produced multiple bands) and 18 clearly were autosomal or X-linked (produced robust PCR amplification in males and females). Note that the accuracy of Y-linkage prediction here (9 out of 36) was well below our previous tests with large or random scaffolds (21 out of 23). The possible justification for these results is explored in the discussion section. Y-linked genes are located in scaffolds GL550523, GL552264, GL552407, GL545474, GL548443, GL551291, GL552021, GL552745 and GL561055 (Table 1).

**Table 1:** Annotation of nine Y-linked genes

Gene	VectorBase annotation	Scaffold	Putative function	Closest Homolog^a^	Nucleotide Identity	Homolog putative linkage
*Met-Y*	RPRC010388	GL552264	Metalloprotease	RPRC007558^c^	72.5 %	A/X
*Zfn-Y1*	RPRC002332	GL552407	Zinc finger protein	*Zfn-Y2*	88.8 %	Y^b^
*Zfn-Y2*	-	GL545474	Zinc finger protein	*Zfn-Y1*	81.5 %	Y^b^
*Aco-Y*	-	GL550523	Aconitate Hydratase/Iron Regulatory Biding Protein	RPRC001246	43.1 %	A/X
*Rpr-Y1*	-	GL548443	Unknown unconserved	KQ034135	97.0 %	AX
*Rpr-Y2*	-	GL551291	Unknown conserved	KQ035043	71.9 %	AX
*Rpr-Y3*	-	GL552021	Unknown conserved	KQ038555	78.8 %	Y
*Rpr-Y4*	-	GL552745	Unknown unconserved	RPRC006814^c^	Not significant	A/X
*Rpr-Y5*	-	GL561055	Unknown unconserved	KQ035385	98.9 %	Undefined

^a^ In the cases where the closest homolog is an annotated gene, we used the VectorBase gene identifier (RPRC000000). Otherwise we used the scaffold number

^b^ Putative linkage confirmed by PCR

^c^ Amino acid identity of 45 % with *Rpr-Y4*

### Structural and functional annotation of Y-linked genes

Our first analysis showed that all nine genes were incomplete and some of them showed premature stop-codons and/or frame-shifts. These problems were expected since Y-linked sequences tend to have sequencing/assembling errors due to shallow coverage [2, 3, 7]. Another frequent assembly problem is that exons of Y-linked genes end up scattered in separated scaffolds due to assembly failure of the repetitive sequences found in introns of heterochromatic genes [2, 3, 10, 11]. Therefore, re-sequencing is usually needed in order to fully annotate Y-linked genes. Sequencing of cDNA was used to close gaps and to correct sequencing errors. Indeed, premature stop-codons and frame-shifts were present in three genes; and cDNA sequencing showed that these were sequencing errors. Rapid amplification of complementary DNA ends (RACE) was used to complete the mRNA sequence of most genes. Y-linked genes were named according to their putative function or as *Rpr-Y(1–5)*, for the genes without previous described function. Table 1 summarizes the annotation status of all nine Y-linked genes.

**Fig. 4:**
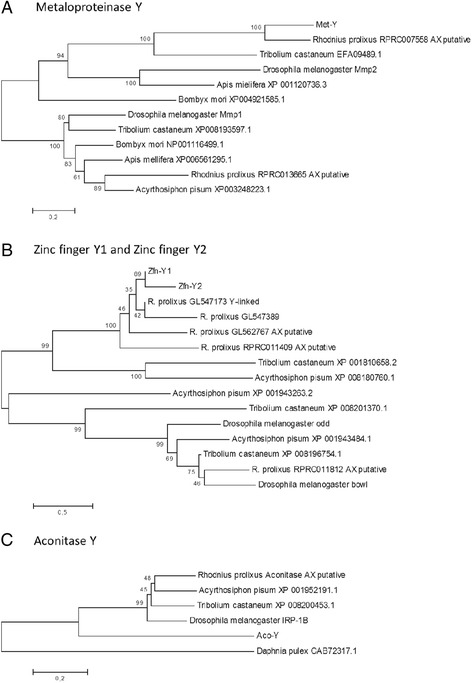
Phylogenetic analysis of Y-linked genes. The phylogenetic trees of genes *Metaloproteinase-Y* (Panel **a**), *Zinc finger-Y1* and *Zinc finger-Y2* (Panel **b**), and *Aconitase-Y* (Panel **c**) are shown. The protein sequences were aligned with ClustalW, and a NJ tree with Poisson correction and complete deletion was constructed with the program MEGA. Bootstrap support values (10000 replicates) are shown inside the tree. We included all sequences returned by the TblastN search. Sequence accession numbers are shown directly in the phylogenetic trees (and can also be found in Table 1 and Additional file 5: Table S3)

**Fig. 5:**
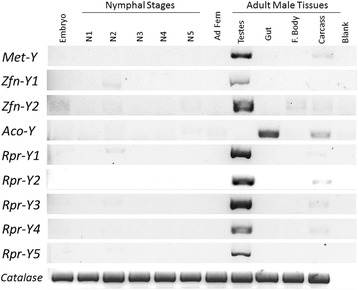
Expression profile of *Rhodius prolixus* Y-linked genes. RT-PCRs were carried out with mRNA purified from embryos (Egg), each nymphal stage (N1 – N5), adult female whole body (Ad Fem), adult male testis, male gut, male fat body (F. Body); and all other male tissues (carcass). A Catalase gene was used as control

Putative function for Y-linked genes was evaluated by both, amino acid identity with known proteins, and phylogenetic analyses of homologs. Four out of nine genes shared homology with proteins with known function. We found an *Aconitase/Iron Regulatory Protein* (*Aco-Y*; accession KP244296), located in scaffold GL550523; a *Metaloprotease* (*Met-Y*; accession KP244297; scaffold GL55264); and two *Zinc Finger Proteins* (*Zfn-Y1* and *Zfn-Y2*; respective accession KP244303 and KP244304; scaffolds GL552407 and GL545474, respectively). Our phylogenetic analysis also suggest that *Met-Y* is orthologous to gene *Mmp2* of *D. melanogaster*, while both zinc finger proteins have orthologous genes in *Tribolium castaneum* (Fig. 4, panels a and b)*.* Our results suggests that *Aco-Y* is not orthologous to the ancestral aconitase of hexapoda (Fig. 4, panel c). The remaining five genes have no similarity to proteins with known functions or motifs; and were named as (*Rpr-Y1*, *Rpr-Y2*, *Rpr-Y3*, *Rpr-Y4* and *Rpr-*Y5; respective accession numbers KP244298, KP244299, KP244300, KP244301 and KP244302). While the gene *Rpr-Y3* has homologues annotated in other genomes (the best match is protein XP008212135.1 of *Nasonia vitripenis*), genes *Rpr-Y1, Rpr-Y2*, *Rpr-Y4* and *Rpr-Y5* are probably *orphan* genes [23] (Table 1). We tested the expression of the nine Y-linked genes in all *R. prolixus* developing stages (embryo, nymphal stages and adults) and for different tissues in the adult male (testis, gut, fat body and carcass; Fig. 5). We found that eight of the nine genes are mostly or only expressed in the testis (genes *Zfn-Y1* and *Rpr-Y5* have expression restricted to testis, while *Met-Y, Zfn-Y2, Rpr-Y2, Rpr-Y2, Rpr-Y3 and Rpr-Y4* presented a strong expression in testis and weak expression in other tissues); whereas *Aco-Y* is mainly expressed in the *R. prolixus* gut (with weak expression in carcass). *Rpr-Y1* also presented a weak expression in the 2^nd^ instar (N2).

### Homologous sequences of Y-linked genes

BlastN and TblastN searches in *R. prolixus* genome and in VectorBase annotated genes revealed that *Rpr-Y4* is a single copy gene (Table 1 and Additional file 5: Table S3). In fact, we found a divergent paralog, RPRC006814 (amino-acid identities of 45 %), for gene *Rpr-Y4. Metaloprotease-Y* and *Aconitase-Y* have only one autosomal or X-linked paralog each (RPRC007558 and RPRC001246 respectively, with respective nucleotide identity of 73 % and 43 %). Phylogenetic analyses of *Met-Y* suggests that this gene is paralogous to RPRC007558 (Fig. 4, panel a). Gene *Rpr-Y2* has three autosomal or X-linked paralogous sequences in the genome (the closest located in scaffold KQ035043, with nucleotide identity of 71 %). We also perceived that that genes *Zfn-Y1*, *Zfn-Y2* are probably a copy of each other (Fig. 4, panel b), with a third copy in the Y scaffold GL547173 (Y-linkage for this scaffold was confirmed by PCR, Additional file 4: Table S2).

Finally, we found 9 homologous sequences for *Rpr-Y1* (nucleotide identities ~97 %), 17 homologous sequences for *Rpr-Y3* (nucleotide identities range from 69 % to 79 %) and 28 homologous sequences for *Rpr-Y5* (nucleotide identities range from 80 % to 99 %). Phylogenetic analyses (Additional file 6: Figure S2.) suggest that *Rpr-Y1* has an autosomal or X-linked paralog (KQ034135). The phylogenetic analyses for *Rpr-Y3* and *Rpr-Y5* (Additional File 6: Figure S2.) suggests that both to a large family of multicopy genes with autosomal/X and Y-linked paralogs.

## Discussion

Studies of Y chromosome genetics started in 1916 when Bridges reported that *Drosophila melanogaster* Y chromosomes were not involved in sex determination, but were essential to male fertility. Almost 30 years later, studies on Klinenfelter and Turner’s Syndromes concluded that the human Y chromosome might have one or more genes, or sectors, which determine the male phenotype. Only in 1993, the mammalian male determining gene (*SRY*) was sequenced [24]. The heterochromatic nature of Y chromosomes hindered most genetic studies of this chromosome. The advance in genomics in 2000 (with the publication of *Drosophila* and Human genome projects) allowed researchers an easy access to the entire genomic data, permitting for faster identification of new Y-linked genes and broader studies of the role of Y chromosomes beyond male sex determination and fertility. Since then, 12 Y-linked genes were described for *Drosophila melanogaster* and a total of 32 genes were described for mammals Y chromosome [8, 10, 11, 25, 26].

The identification of Y-linked genes allowed studies on the evolution of gene content in the Y chromosomes of *Drosophila* and mammals. In *Drosophila*, the research suggested that the gain of genes, instead of loss, plays an important role in Y chromosome evolution [7]. In mammals, recent investigation revealed a low genetic conservation, the importance of gene gain and that Y chromosome may influence male life span [26–28]. Hence, the identification of Y-linked genes is crucial for the understanding of Y chromosome function, origin and evolution.

In 2004, Krzywinski and coworkers [15] proposed that the separate sequencing of male and female genomes would facilitate the identification of Y-linked sequences. Using this method we putatively identified 766 Y chromosome sequences covering more than 1.6 Mb (0.2 % of the genome size). PCR tests for the ten largest candidates showed that all ten are Y-linked; a similar test for another 13 scaffolds (chosen irrespectively to size) found 11 Y-linked and two failures (adding to 21 confirmed Y-linked scaffolds). However, when we tested Y-linkage for 36 genes, we found that only nine candidate scaffolds were Y-linked (for a total of 30 confirmed Y-linked scaffolds). The rate of success for Y-linkage dropped to 31 % (9/27 since nine PCRs failed) when we aimed for genes. Hence, although it is clear that the method of identification of Y-linked sequences proposed by Krzywinski and collaborators works, it had a rather high rate of false positives when applied to the *Rhodnius* genome.

Recently, two independent researchers developed new methods to find Y-linked genes in traditional genome projects (in which male and female DNA are sequenced together) [12, 14]. Both studies used a massive number of Illumina short reads, which provide a high read coverage, increasing confidence to predict Y-linked genes. The method used by Carvalho and Clark (named YGS) used the *Drosophila virilis* high quality virCA assembly (with only 1,186 scaffolds) and ~85 fold coverage of Illumina reads from female DNA. In a similar way, Hall and coworkers searched for Y-linked sequences using a Chromosome Quotient (CQ) method in the *Anopheles gambiae* genome, using ~30 fold coverage of Illumina reads from each sex to calculate probability of Y-linkage. In contrast, the *R. prolixus* assembly has a mean coverage of eight reads/base, which is considered low even for Sanger sequenced genomes (*eg. D. melanogaster* has a mean coverage of 13x [29]). Also, *R. prolixus* genome has 27,872 scaffolds (more than 22,000 of them with less than 5 kb) and almost 2,000 sequences are needed to cover ~90 % of the genome [22]. This comparison suggests that high coverage (and, in a lower scale, assembly quality) is crucial to reduce false positives. The *R. prolixus* low coverage assembly reduces the strength of our approach since the fewer the number of reads used to assemble a scaffold, the higher the chance of such scaffold to be assembled with reads from only one sex.

It is also important to note that the number of false positives increased when we aimed our searches towards protein coding genes. Y chromosomes are remarkably poor in protein coding genes [25, 30]. Therefore, the narrow search for Y-linked genes might have created a bias toward false positives. Also, most candidates are composed of repetitive sequences or multi-copy genes (indeed, six of the nine genes described here have three or more homologs in the genome), and in many cases we had difficulties to design specific primers. Nonetheless, we were able to find nine new Y-linked genes on the *R. prolixus* genome, showing the strength of the method applied here.

Many genomes are now being sequenced using Illumina platform [31, 32]. In genomes assembled from short reads (such as those from Illumina), reads are fragmented in shorter sequences (*k-mers*) [33], and the final assembly does not contain the information of which reads were used to assemble a specific contig (the information we used to find Y candidates). Nevertheless, separate sequencing for each sex (as proposed by Krzywinski [15]), is a very powerful approach in order to enable fast identification of Y-linked sequences in future genomes. In this case, short reads from male and female libraries can be easily aligned in the assembled genome to identify Y-linked sequences (similarly to the CQ method [12]), with the advantage of zero additional cost.

Based on protein similarity and phylogenetic analysis, we were able to attribute putative functions to four Y-linked genes. *Met-Y* gene is a member of the M10 metalloproteases, orthologous to *Drosophila melanogaster Mmp2* protein, which are zinc-dependent endopeptidases, synthesized as inactive precursors [34]. Our results suggest that *Met-Y* expression is restricted to testis. Hence, it is tempting to imagine that *Met-Y* has an important role in male fertility and functional analysis (i.e. expression silencing through RNAi) are being carried out to ascertain this. We also found two zinc finger proteins (*Zfn-Y1* and *Zfn-Y2*) containing a classical C2H2 zinc finger domain. This class of zinc fingers is best known for its role as transcription factors in sequence-specific DNA-binding proteins [35, 36]. Like most of Y-linked genes described here, both zinc finger proteins are mainly expressed in testis. The fourth gene identified is the *Aconitase-Y.* Aconitase is an essential enzyme in the tricarboxylic acid cycle and, interestingly, an iron regulatory protein [37]. Iron regulatory proteins are especially important to hematophagous organisms, since excess of iron could be damaging to cells [38]. In *R. prolixus* females (and other blood feeding insects), iron intake and metabolism is crucial for egg development. However, there are no studies of the iron metabolism in *Rhodnius* males. We found that *Aco-Y* is expressed only in male adult gut (and is the only Y-linked gene described here that is not expressed in testis). The lack of *Aco-Y* expression in other developmental stages and other tissues (other than gut) suggests that *Aco-Y* has a primary function in iron regulation, instead of its role in the tricarboxylic acid cycle (in which case we would expect its expression to be widespread in different developmental stages and tissues).

We have no indication of the biological function of the other six Y-linked genes. While genes *Rpr-Y3* has similar sequences annotated in other genomes, genes *Rpr-Y1*, *Rpr-Y2, RprY-4* and *Rpr-Y5* are probably orphan genes. Similarly to most Y-linked genes in other organisms [8, 12, 27], eight Y-linked genes are expressed mainly in *R. prolixus* testis, in a coherent testis-restricted pattern observed in *Drosophila* [30] and in many human genes [25]. Very little is known about *R. prolixus* sexual differentiation and the role of the Y chromosome in sex determination is still unclear. Sexual dimorphism and gonadal development can only be observed in 5^th^ instar nymphs. Most Y-linked genes described here presented mRNA expression restricted to male adults and, although not impossible, it is very unlikely that any of these have any roles in sex determination. Future functional studies (e.g. quantitative expression analyses and RNAi silencing) are needed for better understanding of the biological role of the *R. prolixus* Y chromosome.

It is very tempting to look at our experimental results and phylogenetic analyses to speculate on the origin and evolution of *R. prolixus* Y-linked genes. The discussion about animals Y chromosome origin and evolution is rich and with different points of view. Although it is widely accepted that Y chromosomes share a common ancestrally with X chromosomes [26, 39, 40], recent studies of *Drosophila* Y chromosomes suggest a non-canonical mechanism of origin and evolution [7, 30, 41]. Our results suggests that some Y-linked genes may have an autosomal or X origin. However, for such discussion, the mapping of X-linked genes is vital to understand Y chromosome origin and evolution. The method used in this study suggests that scaffolds assembled with ~30 % male reads could be X-linked. Further studies (eg. Fluorescent In Situ Hybridization) are needed to ascertain this question. Also, we need the genome analysis of other triatomines to determine the origin of each Y-linked gene, as well as a good genetic map of *R. prolixus* scaffolds to ascertain the location of each paralog. Only afterwards it would be possible to answer the questionings about *R. prolixus* Y chromosome evolution.

All the Y sequences and genes described here could also be used as molecular markers in population studies, which have great importance for vector biology and control research [42–44]. Since these sequences have a male specific non-recombinant inheritance, they are good markers for such studies. In fact, we have already used Y sequences as male specific markers in recent analysis of *R. prolixus* embryogenesis, revealing the usefulness of such sequences [45].

## Conclusions

We have demonstrated that the simple choice, proposed by Krzywinski and coworkers in 2004, of sequencing male and female DNA separately in new genome projects, is of great value for the identification of Y-linked genes. With little computational effort we were able to find hundreds of putative Y-linked sequences. Here we described, for the first time, nine new Y-linked genes in the *Rhodnius prolixus* genome. This methodology is advantageous when compared with classical genome sequencing approaches (where male and female DNA is not separated before sequencing), since it allows the identification of Y-linked sequences at basically zero additional cost. In many genomes, the sequenced organisms have Y chromosomes but these are missed during genome assembling. Great computational and human effort is then needed to unveil Y sequences from such genomes and, in many cases, resequencing is needed to facilitate the process. Although many false positives could arise from low coverage and low quality assemblies, we showed that the method proposed by Krzywinski and coworkers is powerful enough to find Y genes with autosomal duplicates sharing amino nucleotide identities above 98 %. The method also allowed for rapid identification of 30 male-specific markers. A few of these markers were already used in other studies and many of them may be useful for further *R. prolixus* population or vector control studies. Even though we were not able to fully describe the biological role of *R. prolixus* Y chromosome, the functional study of Y-linked genes could reveal important genes for male development, fertility or sex determination. The sequencing of new organisms, especially different triatomines species, applying the same approach as described here for *R. prolixus*, could prove useful for the rapid identification of Y chromosome genes, contributing to further understanding of the evolution and function of animal Y chromosomes.

## Methods

### Computational identification of Y-linked candidates

*Rhodnius prolixus* genome (GenBank: ACPB00000000.3) was assembled using reads from libraries prepared from male or female DNA [22]. Male libraries were named NAAX (Sanger reads) or GFL7EVZ (454 Roche reads) and all reads from such libraries received the NAAX prefix or GFL7EVZ prefix. Female libraries were named NADD, NADK and NADN (no female reads were produced with 454 sequencers) and all reads received prefixes accordingly. A program written in AWK was used to read the *R. prolixus* assembly file and to count the number of reads from male and female libraries used to assemble each contig and scaffold. Numbers were then plotted on a table with: 1) information of scaffold number, 2) scaffold size, 3) number of reads incorporated in assembled scaffold, 4) number of reads from male libraries, 5) number of reads from female libraries and, 6) percentage of male reads in the assembled scaffold. In the *R. prolixus* genome, a total of 4,227,964 male reads (2,818,061 from Sanger and 1,409,903 from 454) and 2,993,635 female reads (corresponding to 58.5 % and 41.5 % respectively) were used in the final assembly. With these numbers we calculated the probability of scaffolds being assembled only with male reads by chance (see Additional file 1: Additional text). All scaffolds assembled with 10 reads or less were excluded from our final analysis, since such scaffolds have more than 1/200 chance to be a false positive (autosomal or X linked scaffolds assembled, by chance, only with male reads). We also excluded from our analysis all scaffolds that were identified as possible bacterial contaminants (presented 100 % identity with bacterial genes in a BLAST search against NR database). For further Y-linkage tests, we selected scaffold GL563091 and GL563092 as our controls, since these scaffolds are clearly autosomal or X-linked (size >12 Mb and ~60 % male reads).

### Identification of Y-linked genes in *Rhodnius prolixus*

All *R. prolixus* scaffolds identified as Y chromosome candidates were softmasked for repetitive sequences using RepeatMasker software. Softmasked scaffolds were blasted against different databases in order to search for gene coding regions. The non-redundant protein (NR), Reference Sequence proteins (Ref-Seq) and bacterial proteins were downloaded from NCBI. Transposable Elements (TE) database was downloaded from RepBase (Repetitive Elements Databse). *Rhodnius prolixus* annotated genes were downloaded from VectorBase. *R. prolixus* transcripts and other identified genes were obtained from the *R. prolixus* genome consortium. We used BLASTx (−W 3, e-value 10^−6^) against NR, Ref-Seq and bacterial proteins. tBLASTx (−W 3, e-value 10^−6^) was used against TE database. BLASTn (Word 28, e-value 10^−8^, ID 100 %) was used against *R. prolixus* transcripts and annotated genes databases. All scaffolds segments that have high similarity (above 95 % nucleotide or amino acid identities) with bacterial proteins or TE databases were discarded from further analysis. Alignments produced from at least one of the other databases were considered as putative Y-linked genes. Putative genes were ordered according to the evidence and we considered the existence of transcripts as the most important evidence of a true codifying Y-linked gene. Other evidences, in order of importance were: 1) alignment with a conserved known gene from Ref-Seq or NR databases; 2) alignment with a conserved hypothetical gene from Ref-Seq database; 3) alignment with a *R. prolixus* annotated gene; 4) alignment with a unconserved hypothetical gene from Ref-Seq or NR databases and; finally, 5) alignment with discarded annotated genes (GeneID_trash) from the *R. prolixus* genome. Genes presenting alignments with at least three different databases (and preferably with the *R. prolixus* transcriptome) were then tested for Y-linkage. Confirmed Y-linked genes were then annotated with GeneWise to identify truncated genes and to define strategies for complete gene annotation. All Y-linked genes without described function were blasted against Protein Family of Domains database (PFam) and Eukariote Conserved Orthologous Database (KOG). Expression of Y-linked genes was then confirmed by RT-PCR (see Molecular biology methods).

### Phylogenetic analysis of Y-linked genes

The evolutionary history of each Y-linked gene were conducted in MEGA5 [46] and inferred using the Neighbor-Joining method. The percentage of replicate trees in which genes clustered together in the bootstrap test (10000 replicates) are shown next to the branches. Evolutionary distances were computed using the Poisson correction method and are in the units of the number of amino acid substitutions per site. Homologous sequences were obtained from VectorBase and from NCBI, and all accession numbers are shown on each tree branch or in the Additional file 5: Table S3.

### Molecular biology methods

Male and virgin female *R. prolixus* genomic DNA was isolated using DNeasy Blood & Tissue Kit (Qiagen, cat# 69504). PCR for the detection of Y-linkage was performed with GoTaq® Hot Start Polymerase (Promega, cat# M5005) and primers design targeted exons (for gene Y-linkage tests) or elsewhere in the scaffold (for scaffold Y-linkage tests). RNA was isolated with TRIzol® Reagent (Invitrogen, cat# 15596–018), following manufacturer instruction from pools of five individuals and for different developmental stages and tissues (embryos, all five instar stages, female whole body, male testis, male gut, male fat body and male carcass). cDNA was synthesized using High-Capacity cDNA Reverse Transcription Kit (Life Technologies, cat# 4368814). Gene expression was evaluated by RT-PCR using the same primers and protocol designed for Y-linkage tests. Rapid Amplification of cDNA ends (Invitrogen, cat# 18373–019 and 18374–058) and RT-PCR (Invitrogen, cat# 12574–035) were performed for gene annotation and nucleotide sequencing correction. All PCR products were Sanger sequenced at Macrogen (Korea).

## Availability of supporting data

Supporting information has been provided as supplementary files to this submission

## Additional files

Additional file 1:
**Additional text.** Supporting material and methods. (PDF 18 kb) Additional file 2: Table S1. Expected false positives per number of reads. (PDF 15 kb) Additional file 3: Figure S1. Y-linkage PCR tests for 13 random scaffolds. (PDF 47 kb) Additional file 4: Table S2. List of confirmed Y-linked scaffolds. (PDF 27 kb) Additional file 5: Table S3. Annotated and non-annotated homologs of each Y-linked gene. (PDF 60 kb) Additional file 6: Figure S2. Phylogenetic analysis of Y-linked genes. (PDF 377 kb)
