# Supplementary material for: First report of Y-linked genes in the kissing bug Rhodnius prolixus
Source: BMC Genomics. 2016 Feb 9;17:100. doi: 10.1186/s12864-016-2425-8 (PMC4746886; doi:10.1186/s12864-016-2425-8)
Supplement: Additional file 1: — Additional text. Supporting material and methods. (PDF 18 kb) [file 12864_2016_2425_MOESM1_ESM.pdf]

Additional text. Supporting material and methods.

Rhodnius prolixus libraries description.

Male libraries were named NAAX (Sanger reads) or GFL7EVZ (454 Roche reads) and all reads from such libraries received the NAAX prefix or GFL7EVZ prefix. Female libraries were named NADD, NADK and NADN (no female reads were produced with 454 sequencers) and all reads received prefixes accordingly.

Calculating the probability of false positives in Y-linkage prediction.

The Assembly RproC1 of the *Rhodnius prolixus* genome contains a total of 4,227,809 traces from male libraries and 2,993,480 traces from female libraries, corresponding to 58.5% and 41.5% of male and female traces respectively. This difference could produce a bias toward the number of male and female traces used to assemble each scaffold, increasing the number of scaffolds assembled only with male traces. To access this bias effect, and the expected number of scaffolds assembled only with male traces by chance (false Y candidates), we calculated the probability of a scaffold assembled with a given number of traces (n) to be assembled only with male traces using the formula $f(m)^n$ (where $f(m)$ is the frequency of male traces in library and n is the number of traces used to assemble a given scaffold.

Then, based on the number of scaffolds assembled with a given number of traces, we calculated the expected number of false positives. Results were plotted in the S1 Table.

S1 Table. Expected false positives per number of traces.

Num of Traces	Only M traces by chance	N of Scaffolds built with the given number of traces	Expected False Positives
2	0,342764583	910	311,9
3	0,200675295	2058	413,0
4	0,117487559	2565	301,4
5	0,068784384	2614	179,8
6	0,040270574	2402	96,7
7	0,023576851	2038	48,0
8	0,013803327	1815	25,1
9	0,008081309	1476	11,9
10	0,004731291	1216	5,8
11	0,002769987	956	2,6
12	0,001621719	818	1,3
13	0,000949453	639	0,6
14	0,000555868	497	0,3
15	0,000325439	433	0,1
16	0,000190532	343	0,1
17	0,000111549	302	0,0
18	6,53076E-05	250	0,0
19	3,8235E-05	217	0,0
20	2,23851E-05	175	0,0
