# Supplementary material for: First report of Y-linked genes in the kissing bug Rhodnius prolixus
Source: BMC Genomics. 2016 Feb 9;17:100. doi: 10.1186/s12864-016-2425-8 (PMC4746886; doi:10.1186/s12864-016-2425-8)
Supplement: Additional file 2: Table S1. — Expected false positives per number of reads. (PDF 15 kb) [file 12864_2016_2425_MOESM2_ESM.pdf]

Additional table 1. Expected false positives per number of traces.

**Num of Traces**	**Only M traces by chance**	**N of Scaffolds built with the given number of traces**	**Expected False Positives**
2	0.342764583	910	311.9
3	0.200675295	2058	413.0
4	0.117487559	2565	301.4
5	0.068784384	2614	179.8
6	0.040270574	2402	96.7
7	0.023576851	2038	48.0
8	0.013803327	1815	25.1
9	0.008081309	1476	11.9
10	0.004731291	1216	5.8
11	0.002769987	956	2.6
12	0.001621719	818	1.3
13	0.000949453	639	0.6
14	0.000555868	497	0.3
15	0.000325439	433	0.1
16	0.000190532	343	0.1
17	0.000111549	302	0.0
18	6.53076E-05	250	0.0
19	3.8235E-05	217	0.0
20	2.23851E-05	175	0.0
