# Supplementary material for: First report of Y-linked genes in the kissing bug Rhodnius prolixus
Source: BMC Genomics. 2016 Feb 9;17:100. doi: 10.1186/s12864-016-2425-8 (PMC4746886; doi:10.1186/s12864-016-2425-8)
Supplement: Additional file 3: Figure S1. — Y-linkage PCR tests for 13 random scaffolds. (PDF 47 kb) [file 12864_2016_2425_MOESM3_ESM.pdf]

Additional figure 1. Y-linkage PCR tests for 13 random scaffolds.

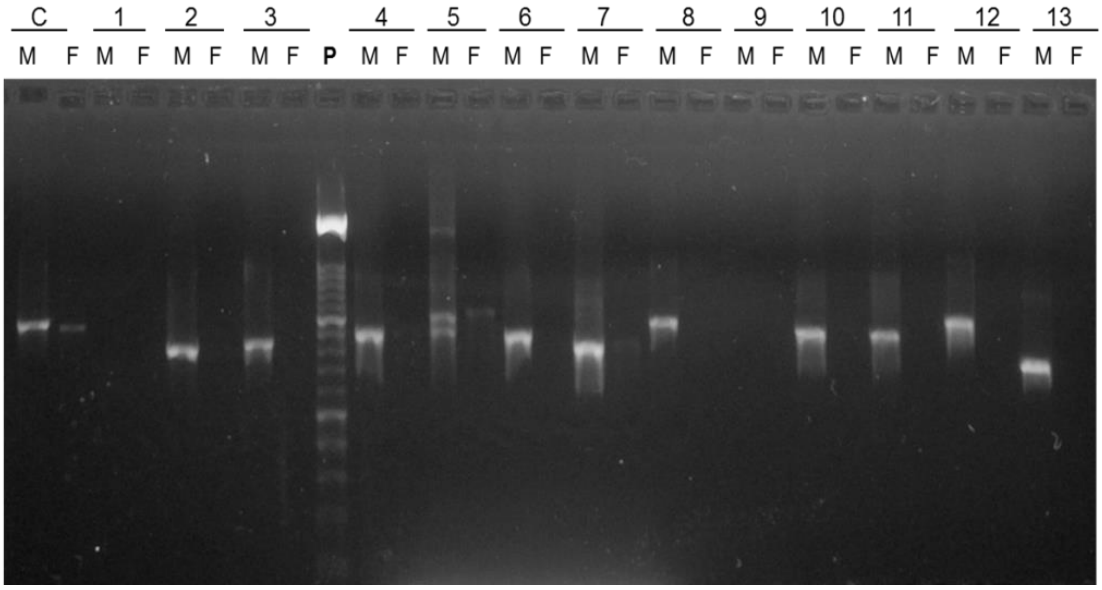

Additional figure 1. Scaffold linkage tests for the *Rhodinius prolixus* Y-chromosome.

Y-linkage was confirmed by PCR. A male specific band implies Y-chromosome linkage. We tested the 13 random Y-candidates and confirmed Y-linkage for 11 of them (two PCRs failed). Primers targeting scaffold GL563092.1 (62% male traces) were used as control. Scaffold numbers are: 1- ACPB03034961; 2- GL547860; 3- KQ037847; 4-GL569928; 5- GL550258; 6- GL564877; 7- GL563586; 8- KQ037143; 9- KQ037150; 10- GL563586; 11- ACPB03034578; 12- ACPB03034456; 13- GL550726
