# Supplementary material for: First report of Y-linked genes in the kissing bug Rhodnius prolixus
Source: BMC Genomics. 2016 Feb 9;17:100. doi: 10.1186/s12864-016-2425-8 (PMC4746886; doi:10.1186/s12864-016-2425-8)
Supplement: Additional file 4: Table S2. — List of confirmed Y-linked scaffolds. (PDF 27 kb) [file 12864_2016_2425_MOESM4_ESM.pdf]

Additional table 2. List of confirmed Y-linked scaffolds.

**Scaffold**	**Size (bp)**	**N of male traces**	**N of female traces**	**Putative genes**	**Confirmed genes**
GL545474	2634	13	0	Zfn-Y2	Zfn-Y2
GL552407	2486	16	0	Zfn-Y1	Zfn-Y1
GL561055	2442	28	0	Rpr-Y5	Rpr-Y5
GL552745	1939	17	0	Rpr-Y4	Rpr-Y4
GL552021	3691	24	0	Rpr-Y3	Rpr-Y3
GL551291	3140	35	0	Rpr-Y2	Rpr-Y2
GL548443	1183	7	0	Rpr-Y1	Rpr-Y1
GL552264	2797	16	0	Met-Y	Met-Y
GL550523	2948	44	0	Aconitase-Y	Aconitase-Y
GL547173	15238	77	0		
GL547860	4452	31	0		
GL548125	7111	26	0		
GL550228	6714	53	0	DNA-Pol	
GL550258	3729	18	0		
GL550726	3595	35	0	t-RNA-Leu	
GL551031	6737	96	0	Transketolase	
GL552288	6514	87	0		
GL552313	7758	39	0		
GL556091	7195	47	0		
KQ037143	4411	55	0	Transmembrane	
GL563586	1274	6	0		
KQ037847	3192	38	0		
GL559384	6583	77	0		
GL559926	7435	85	0	Transmembrane14C	
GL561532	5997	73	0		
ACPB03034578	4853	40	0	Transmembrane	
ACPB03034456	4952	38	0		
GL563586	1274	6	0		
GL564877	1214	11	0		
GL569928	1348	14	0	DNA/Protein translocase	
