# Supplementary material for: First report of Y-linked genes in the kissing bug Rhodnius prolixus
Source: BMC Genomics. 2016 Feb 9;17:100. doi: 10.1186/s12864-016-2425-8 (PMC4746886; doi:10.1186/s12864-016-2425-8)
Supplement: Additional file 5: Table S3. — Annotated and non-annotated homologs of each Y-linked gene. (PDF 60 kb) [file 12864_2016_2425_MOESM5_ESM.pdf]

Additional table 3. Annotated and non-annotated homologs of each Y-linked gene.

Gene	Annotated Homologs	Nucleotide Identity	Homolog putative linkage	Non-annotated genome Homologs	Nucleotide Identity	Homolog putative linkage
Met-Y	**RPRC007558**	**72.5%**	**AX**	None	-	
Zfn-Y1* and *Zfn-Y2	RPRC011409	72.8%	AX	**GL547173**	**89.6%**	**Y (confirmed)**
	RPRC006245	75.3%	Undefined	GL550493	73.9%	Y
	RPRC007834	73.5%	Undefined	GL562767	75.7%	AX
				GL547389	88.8%	Undefined
				GL549943	74.0%	Undefined
Aco-Y	RPRC001246	43.1%	AX	None	-	
Rpr-Y1	None	-		**KQ034135**	**97.0%**	**AX**
				KQ034147	96.8%	AX
				KQ034805	96.8%	AX
				KQ034982	96.8%	AX
				KQ034138	96.6%	AX
				GL562927	96.6%	AX
				GL560954	96.6%	AX
				**ACPB03040363**	**97.4%**	**Undefined**
				ACPB03041068	97.0%	Undefined
Rpr-Y2	RPRC006814	Not Significant	AX	**KQ035043**	**71.9%**	**AX**
				KQ036266	67.5%	AX
Rpr-Y3	RPRC004151	Not significant	Y	**KQ038555**	**78.8%**	**Y**
				ACPB03043895	73.0%	Y
				ACPB03037508	72.3%	Y
				GL547858	70.4%	Y
				KQ036074	75.5%	AX
				KQ035043	74.9%	AX
				KQ037191	74.7%	AX
				KQ035850	78.5%	Undefined
				KQ035850	78.2%	Undefined
				KQ038414	74.4%	Undefined
				GL558838	73.9%	Undefined
				GL554222	73.3%	Undefined
				GL546657	72.2%	Undefined
				GL545715	71.6%	Undefined
				GL563294	70.9%	Undefined
				GL549847	69.4%	Undefined
Rpr-Y4	**RPRC006814**	**Not significant**	**AX**	None	-	
Rpr-Y5	None	-		ACPB03038183	90.5%	Y
				KQ039174	86.4%	Y
				ACPB03037791	85.6%	Y
				KQ035850	85.3%	Y
				KQ034167	85.2%	Y
				KQ035918	85.2%	Y
				ACPB03047459	84.8%	Y
				KQ034178	92.4%	AX
				ACPB03040329	86.3%	AX
				KQ036222	86.2%	AX
				KQ039086	85.9%	AX
				ACPB03037829	85.6%	AX
				KQ038262	85.2%	AX
				KQ037814	85.2%	AX
				KQ035385	98.9%	Undefined
				KQ038266	92.4%	Undefined
				ACPB03045140	86.7%	Undefined
				ACPB03038530	86.4%	Undefined

				ACPB03042646	86.3%	Undefined
				ACPB03037988	86.2%	Undefined
				ACPB03043362	85.8%	Undefined
				KQ034341	85.2%	Undefined
				GL567658	84.8%	Undefined
				GL560296	84.8%	Undefined
				GL550597	84.4%	Undefined
				GL556562	84.3%	Undefined
				GL556476	81.2%	Undefined
				GL548076	80.4%	Undefined
