# Supplementary material for: First report of Y-linked genes in the kissing bug Rhodnius prolixus
Source: BMC Genomics. 2016 Feb 9;17:100. doi: 10.1186/s12864-016-2425-8 (PMC4746886; doi:10.1186/s12864-016-2425-8)
Supplement: Additional file 6: Figure S2. — Phylogenetic analysis of Y-linked genes. (PDF 377 kb) [file 12864_2016_2425_MOESM6_ESM.pdf]

Additional Figure 2. Phylogenetic analysis of five *R. prolixus* Y-linked genes

Panel A – Rpr-Y1

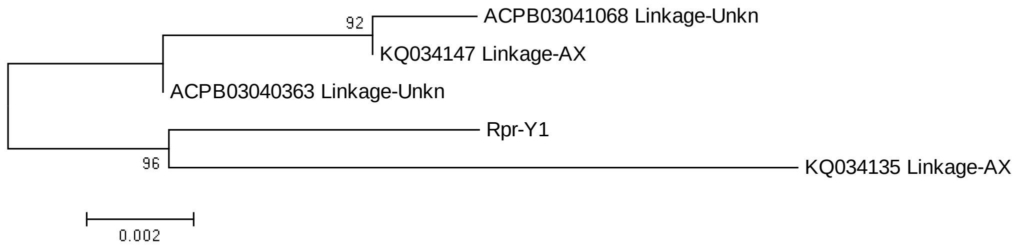

Panel B – Rpr-Y2

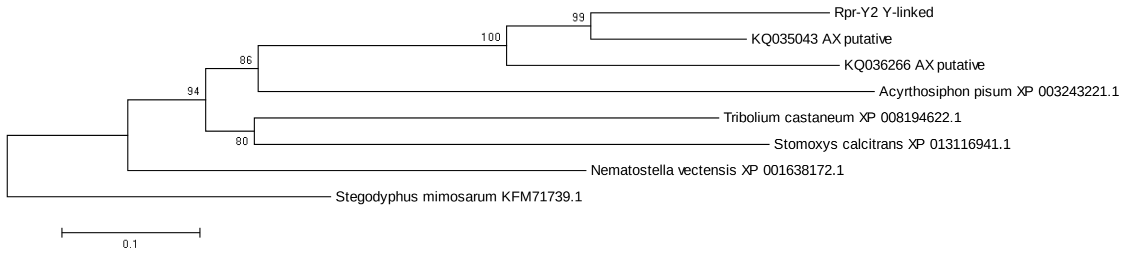

Panel C – Rpr-Y3

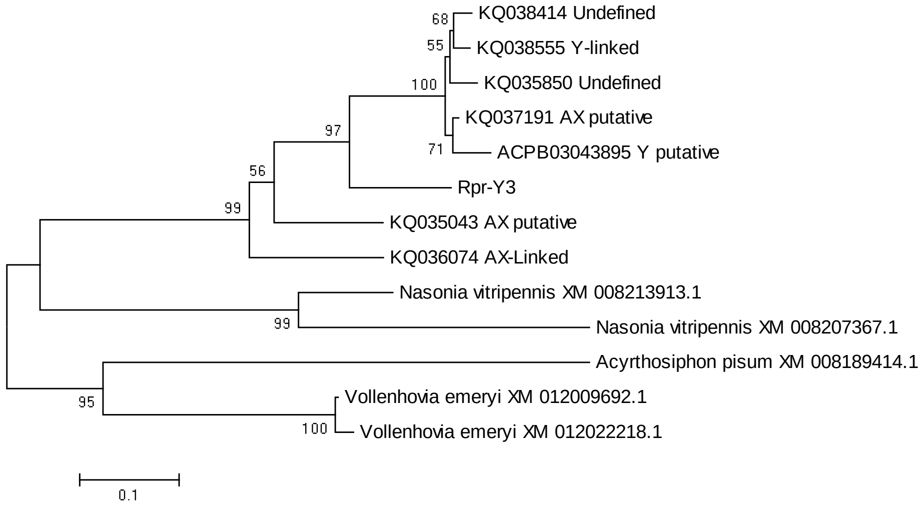

Panel D – Rpr-Y4

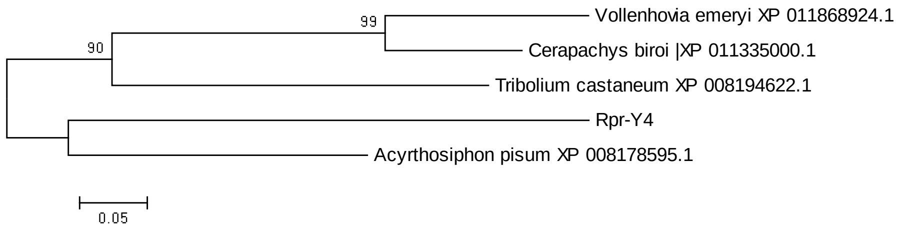

Panel E – Rpr-Y5

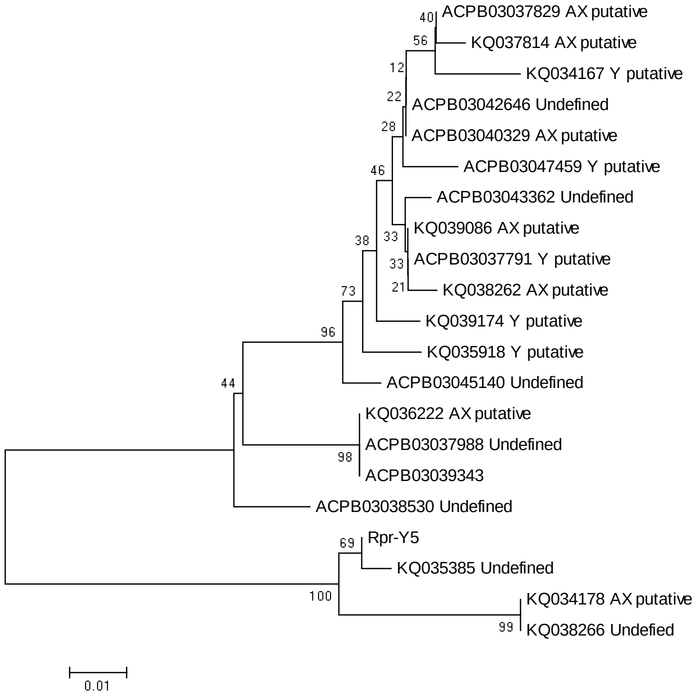

Additional Figure 2 – Evolutionary analysis of five Y-linked genes by phylogeny. The protein sequences were aligned with ClustalW, and a NJ tree with Poisson correction and complete deletion was constructed with the program MEGA. Each panel shows the phylogenetic analysis of one gene, indicated in the top of the corresponding panel. We included all sequences returned by the TblastN search. Sequence accession numbers are shown directly in the phylogenetic trees (and can also be found in Table 1 and Additional table 3).
